# Long‐term exposure to triclosan increases migration and invasion of human breast epithelial cells in vitro

**DOI:** 10.1002/jat.4097

**Published:** 2020-11-10

**Authors:** Abdullah Farasani, Philippa D. Darbre

**Affiliations:** ^1^ Biomedical Research Unit, Medical Research Centre, and Department of Medical Laboratory Technology, College of Applied Medical Sciences Jazan University Jazan Saudi Arabia; ^2^ School of Biological Sciences University of Reading Reading UK

**Keywords:** breast cancer, E‐cadherin, invasion, migration, motility, triclosan, xCELLigence

## Abstract

Extensive use of triclosan (2,4,4′‐trichloro‐2′‐hydroxydiphenyl ether) as an antimicrobial agent in household and personal care products has resulted in global exposure of the human population. Its presence in human tissues, including milk, and its oestrogen‐disrupting properties raise concerns for an involvement in breast cancer. Because metastatic tumour spread is the main cause of breast cancer mortality, we have investigated the effects of triclosan on cell migration and invasion using three human breast epithelial cell lines and using concentrations comparable with those in human tissues. Long‐term exposure to 10^−7^ M of triclosan resulted in increased migration and invasion as measured by xCELLigence technology for all three cell lines, for the immortalized but nontransformed MCF‐10F breast epithelial cells (after 28 weeks), the oestrogen‐responsive MCF‐7 breast cancer cells (after 17 weeks) and the oestrogen‐unresponsive MDA‐MB‐231 breast cancer cells (after 20 weeks). The effects were therefore not limited to cancerous cells or to oestrogen‐responsive cells. This was paralleled in the MCF‐10F and MCF‐7 (but not MDA‐MB‐231) cells by a reduction in levels of E‐cadherin mRNA as measured by reverse transcription–polymerase chain reaction (RT‐PCR) and of E‐cadherin protein as measured by western immunoblotting, suggesting a mechanism involving epithelial‐to‐mesenchymal transition. This adds triclosan to the increasing list of ingredients of personal care products that can not only enter human breast tissue and increase cell proliferation but also influence cell motility. If mixtures of components in household and personal care products contribute to increasing cell migration and invasion, then reduction in exposure could offer a strategy for reducing breast cancer spread.

## Abstract 2

Long‐term (>15 weeks) exposure to 10^−7^ M of triclosan resulted in increased migration and invasion of three human breast epithelial cell lines as measured using xCELLigence technology. Effects were not limited to cancerous cells or to oestrogen‐responsive cells. In two cell lines, reduced levels of E‐cadherin (mRNA and protein) were observed, suggesting a mechanism involving epithelial‐to‐mesenchymal transition. Triclosan is therefore another ingredient of personal care products that is entering the human breast and increasing cell motility, which is a prerequisite for metastasis.

## 1 INTRODUCTION

**FIGURE 1:**
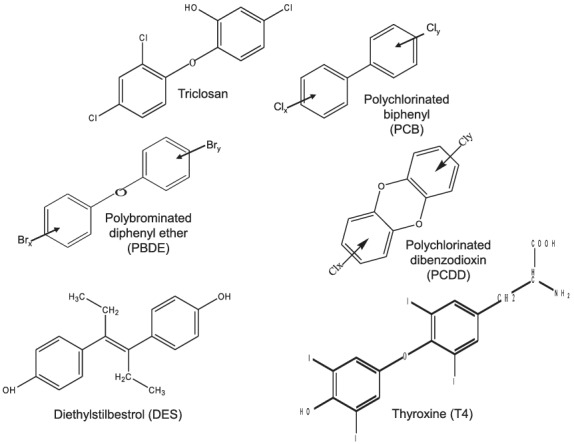
Chemical structure of triclosan (CAS number 3380‐34‐5) compared with the structures of other environmental halogenated aromatic compounds, the polychlorinated biphenyls (PCBs), the polychlorinated dibenzodioxins (PCDD) and the polybrominated diphenyl ethers (PBDE). Comparisons with the structures of the synthetic oestrogen, diethylstilbestrol (DES) and thyroxine (T4) are also shown

The human population is extensively exposed to triclosan (2,4,4′‐trichloro‐2′‐hydroxydiphenyl ether) through its widespread use as an antimicrobial agent in consumer goods (Halden et al., 2017). It was originally used as a hospital scrub in the 1970s but over time became incorporated into many household and personal care products (Halden et al., 2017). Its addition to personal care products such as hand sanitizers, liquid soaps, toothpaste and mouthwashes (Halden et al., 2017) remains under regulatory review in the United States by the Food and Drug Administration (FDA) (www.fda.gov) and in the European Union by the European Chemicals Agency (ECHA) (echa.europa.eu) (SCCP, 2009). The chemical structure of triclosan shows that it is a polychlorinated aromatic compound, not dissimilar to other polyhalogenated organic compounds now restricted for their toxicity and environmental stability, such as the polychlorinated biphenyls, polybrominated diphenyl ethers and polychlorinated dibenzodioxins (Figure 1). Similarities in structure to the synthetic oestrogen, diethylstilboestrol (DES) and the thyroid hormone (thyroxine) (Figure 1) may explain its reported endocrine‐disrupting properties (Halden et al., 2017).

Widespread use of triclosan‐containing consumer products has resulted in the detection of triclosan across the ecosystem in all corners of the globe in water (wastewater, sewage, rivers) and in tissues of aquatic organisms (Dann & Hontela, 2011). It has been reported as a component of dust in private homes in Spain (Canosa, Rodriguez, Rubi, & Cela, 2007) reflecting its widespread use in the domestic environment. The human population may be exposed by multiple routes: dermal absorption from application of personal care products, absorption from the oral cavity and/or ingestion of products such as toothpaste or mouthwash and inhalation from aerosols or indoor dust. Triclosan has been measured extensively in human tissues indicating its systemic distribution in the human body. Triclosan has been detected in human plasma (Allmyr, Adolfsson‐Erici, McLachlan, & Sandborgh‐Englund, 2006; Allmyr et al., 2008; Allmyr, McLachlan, Sandborgh‐Englund, & Adolfsson‐Erici, 2006; Hovander et al., 2002; Sandborgh‐Englund, Adolfsson‐Erici, Odham, & Ekstrand, 2006) and urine (Calafat, Ye, Wong, Reidy, & Needham, 2008; Heffernan et al., 2015; Provencher et al., 2014; Yin et al., 2016); 75% of the U.S. population sampled between 2003 and 2004 had triclosan in urine at concentrations up to 13.1 μM (Calafat et al., 2008). In China, 80% of study participants had triclosan in their urine (Yin et al., 2016). In Australia, of 2,400 urine samples taken in 2012–2013, all had triclosan and at levels ranging from 0.08 to 0.71 μM (Heffernan et al., 2015). Other studies have measured triclosan in the urine of pregnant women (Meeker et al., 2013; Mortensen et al., 2014; Pycke et al., 2014; Weiss et al., 2015) and of children (Wolff et al., 2007). Further studies demonstrate its presence in the amniotic fluid (Philippat et al., 2013; Shekhar et al., 2017), cord blood (Pycke et al., 2014) and even fingernails/toenails (Yin et al., 2016). Studies of human adipose tissue demonstrate its presence with a geometric mean of 7.21 ng/g wet weight of tissue (Wang, Asimakopoulos, & Kannan, 2015). The detection of triclosan in human breast milk (Adolfsson‐Erici, Pettersson, Parkkonen, & Sturve, 2002; Allmyr, Adolfsson‐Erici, et al., 2006; Allmyr, McLachlan, et al., 2006) suggests the potential for adverse effects in babies at a sensitive life stage (Dayan, 2007). Correlation analyses have demonstrated associations between levels of triclosan present in plasma and milk and the use of specific personal care products containing triclosan (Allmyr, McLachlan, et al., 2006). Swallowing one tablespoon of mouthwash was shown to raise plasma triclosan levels up to 1 μM within 1–3 h (Sandborgh‐Englund et al., 2006). Triclosan in products applied to the skin could enter through dermal absorption, and triclosan has been shown to be absorbed readily through human skin (Moss, Howes, & Williams, 2000), where it may be retained (Manevski et al., 2015).

Triclosan is being increasingly linked with a range of adverse human health effects (Weatherly & Gosse, 2017), but the presence of triclosan in milk (Adolfsson‐Erici et al., 2002; Allmyr, Adolfsson‐Erici, et al., 2006; Allmyr, McLachlan, et al., 2006), which implies transfer from the human breast, raises the potential for a possible role in breast cancer development (Darbre, 2015). Triclosan has been shown to possess endocrine‐disrupting properties in the aquatic ecosystem (Dann & Hontela, 2011) and more specifically to interfere with oestrogen action in a rodent model in vivo (Stoker, Gibson, & Zorrilla, 2010) and in assay systems in vitro (Ahn et al., 2008; Gee, Charles, Taylor, & Darbre, 2008; Henry & Fair, 2013; Huang et al., 2014). Oestrogen is a main risk factor for breast cancer development (Miller, 1996), and the oestrogenic properties of triclosan may contribute further to the oestrogenic burden of the human breast (Darbre, 2019). Other oestrogenic constituents of personal care products that may also contribute include the alkyl esters of *p*‐hydroxybenzoic acid (parabens) used as preservatives, some of the chemical UV filters, aluminium salts used as antiperspirant, some components of fragrance and components of conditioning such as the cyclic volatile methylsiloxanes (cVMS) (Darbre, 2019). All these chemicals, however, possess other adverse properties in addition to the endocrine‐disrupting activities, one of which is their ability to increase motility of human breast cancer cells. Previous work has shown that long‐term exposure to parabens (Khanna, Dash, & Darbre, 2014), chemical UV filters (Alamer & Darbre, 2018) or aluminium salts (Bakir & Darbre, 2015; Darbre, Bakir, & Iskakova, 2013) can increase migration and invasion of oestrogen‐responsive breast cancer cells. Because 17β‐oestradiol is known to increase cell motility (Li et al., 2010; Planas‐Silva & Waltz, 2007; Sanchez et al., 2010), these chemically induced responses could result from the oestrogenic activity of these chemicals. However, other mechanisms must also exist because some effects were also observed in oestrogen‐unresponsive cells (Alamer & Darbre, 2018; Bakir & Darbre, 2015). This is important because increases in migration and invasion of cancer cells are essential components of metastasis (Scheel & Weinberg, 2012), which is the main cause of mortality for breast cancer (Miller, 1996). One previous publication has reported increased migration and invasion of oestrogen‐responsive MCF‐7 human breast cancer cells following short‐term (up to 72 h) exposure to 10^−6^ M of triclosan under oestrogen‐deprived conditions (Lee, Choi, & Hwang, 2017). We report here effects on motility of breast epithelial cells over a longer, more environmentally relevant time course (up to 30 weeks), using lower and more environmentally relevant concentrations (10^−7^ M) and in the presence of oestradiol, using oestrogen‐unresponsive as well as oestrogen‐responsive breast cancer cells and using nontransformed as well as transformed breast epithelial cells.

## 2 MATERIALS AND METHODS

### 2.1 Materials

Triclosan (2,4,4′‐trichloro‐2′‐hydroxydiphenyl ether) (CAS number 3380‐34‐5) (5‐chloro‐2‐(2,4‐dichloro‐phenoxy)phenol (IrgasanDP300) was a gift from CIBA (Macclesfield, England). 17β‐Oestradiol was purchased from Steraloids (Croydon, UK). Stock solutions of triclosan and 17β‐oestradiol were made in ethanol and diluted 1 in 10,000 (v/v) into culture medium: controls contained equivalent concentrations of ethanol.

### 2.2 Stock culture of human breast cells

MCF‐10F human breast epithelial cells (Soule et al., 1990) were purchased from the American Tissue Culture Collection at passage number 110. These cells are considered as immortalized but not transformed (Soule et al., 1990). The cells were maintained as monolayer cultures in a stock culture medium containing 1:1 (v/v) Dulbecco's modified Eagle's medium (DMEM) and Ham's F12 (Invitrogen) supplemented with 5% (v/v) horse serum (Invitrogen), 500 ng/ml of hydrocortisone (Sigma), 10 μg/ml of insulin (Sigma) and 20 ng/ml of epidermal growth factor (Sigma) in a humidified atmosphere of 10% carbon dioxide in air at 37°C.

MCF‐7 human breast cancer cells were provided by Osborne in 1987 at passage number 390 from the laboratory of Charles McGrath (Osborne, Hobbs, & Trent, 1987). These cells remain dependent on oestrogen for proliferation when maintained as monolayer cultures in DMEM (containing phenol red) (Invitrogen, Paisley, UK) supplemented with 5% (v/v) foetal calf serum (Invitrogen), 10 μg/ml of insulin (Sigma‐Aldrich) and 10^−8^ M of 17β‐oestradiol (Steraloids, Croydon, England) in a humidified atmosphere of 10% CO_2_ in air at 37°C (Darbre & Daly, 1989; Shaw, Sadler, Pugazhendhi, & Darbre, 2006).

MDA‐MB‐231 human breast cancer cells were purchased from the American Tissue Culture Collection at passage number 28. These cells are not considered as responsive to oestrogen for growth (Garcia, DeRocq, Freiss, & Rochefort, 1992). The cells were maintained as monolayer cultures in DMEM (containing phenol red) (Invitrogen) supplemented with 10% (v/v) foetal calf serum (Invitrogen) in a humidified atmosphere of 10% carbon dioxide in air at 37°C.

All cell stocks were sub‐cultured at weekly intervals by suspension with 0.06% trypsin, 0.02% EDTA pH 7.3 and re‐plating the cells at a density of 0.2 × 10^5^ cells/ml in 16‐ml aliquots in 9‐cm tissue culture dishes, and medium was replenished after 3–4 days.

### 2.3 Long‐term growth of human breast cells with triclosan

Long‐term exposure of cells to triclosan was carried out for all cell lines using the stock media as described above and simply growing parallel cultures with or without the addition of triclosan. Triclosan was added at 10^−7^ M, this having been the pre‐determined maximal concentration without adverse effects on proliferation rate of any of the cell lines used here. Triclosan was made up in ethanol and diluted 1 in 10,000 (v/v) into culture medium. Controls contained the same volume of ethanol.

Although triclosan has some oestrogenic activity (Gee et al., 2008) and some of the cell lines show oestrogen responsiveness (MCF‐7 and MCF‐10F—see above), the effects of oestrogen deprivation (Shaw et al., 2006) were not studied in these experiments. The cells were grown simply in their stock media in order to focus the study on triclosan effects alone. Cells were subcultured at weekly intervals as described above.

### 2.4 Assay of cell migration and invasion using xCELLigence technology

The CIM‐plate 16 contains 16 wells, each a modified Boyden chamber, which can be used independently but simultaneously to measure cell migration in real‐time through 8‐μm pores of a polyethylene terephthalate membrane onto gold electrodes on the underside of the membrane using the xCELLigence system (ACEA Biosciences, San Diego, CA, USA). Experiments were set up according to the manufacturer's instructions with the membrane uncoated (migration) or coated with growth‐factor‐reduced‐matrigel (invasion) (BD Biosciences, Oxford, UK) (20 μl 1:40 diluted matrigel per well on the upper surface). A chemotactic signal for movement was provided by inoculating the cells in stock medium lacking serum in the upper chamber (with 10^−7^ M of triclosan or ethanol control as appropriate) and supplying the complete stock medium with serum in the lower chamber (with 10^−7^ M of triclosan or ethanol control as appropriate). Cell index (electrical impedance) was monitored every 15 min for the duration of the experiment. Traces show the average of quadruplicate wells.

### 2.5 Real‐time RT‐PCR

Following growth of stock cells for the indicated period of time with or without 10^−7^ M triclosan (see above), cells were plated at a density of 12.8 × 10^5^ cells per 9‐cm culture dish (16 ml) and grown for 7 days in stock medium continuing with or without the respective concentration of triclosan. This enabled cells to be harvested (using a rubber policeman) at similar densities, and whole cell RNA was prepared using the RNeasy mini kit with a Qiashredder column according to manufacturer instructions (Qiagen). First strand cDNA synthesis was performed using the QuantiTect reverse transcription kit (Qiagen) and polymerase chain reactions were carried out using the QuantiTect SYBER Green PCR kit (Qiagen) together with E‐cadherin (CDH1) (QT00080143), β‐catenin (CTNNB1) (QT00077882), or β‐actin (QT01680476) QuantiTect primers (Qiagen) according to manufacturer protocols. Quantitative values were obtained from the threshold cycle (C_T_) number at which the exponential increase in fluorescent signal from the PCR product was at the midpoint (50%). All reactions were performed in triplicate. The C_T_ number for each gene target (E‐cadherin or β‐catenin) was normalized to the C_T_ number for the corresponding β‐actin reaction and values from cells grown with triclosan normalized to control values of cells grown without triclosan using the 2^−ΔΔC^_T_ method of relative gene expression analysis (Livak & Schmittgen, 2001). Average values for the triplicate reactions were then calculated. This was then repeated for each of the three biological replicates generated from independent cell cultures, and results presented show the average ± standard deviation (SD) (*n* = 3) of the three biological replicates. According to the 2^−ΔΔC^_T_ method, results are presented relative to the control value of 1.0 for cells grown in the absence of triclosan.

### 2.6 Western immunoblotting

Cells were grown and lysates prepared as described previously (Shaw et al., 2006) using lysis buffer (50‐mM Tris‐HCl pH 7.4, 250‐mM NaCl, 5‐mM EDTA, 0.3% [v/v] Triton‐X‐100, 4‐(2‐aminoethyl)benzenesulfonyl fluoride [AEBSF 0.3 mM], leupeptin [10 μg/ml] and aprotonin [2 μg/ml]). Lysates were incubated on ice 30 min, passed through needles 19G–25G, sonicated for 2 s and run on 10% sodium dodecyl sulfate‐polyacrylamide gel electrophoresis (SDS–PAGE) Bio‐Rad stain‐free gels (25‐μg protein per track), and proteins were transferred onto Bio‐Rad polyvinylidene difluoride (PVDF) membranes using the Bio‐Rad Trans‐Blot‐Turbo semi‐dry transfer system according to manufacturer's instructions (Bio‐Rad, Watford, UK). Membranes were blocked and immunoblotted as published (Shaw et al., 2006) but using Tris‐buffered saline (TBS). Primary antibodies to E‐cadherin (3195; 24E10) (Cell Signaling, New England Biolabs, Hitchin, UK) were diluted 1/1,000; primary antibody to β‐actin (Cell Signaling) was diluted 1/5,000; horseradish peroxidase (HRP)‐linked secondary antibodies (Cell Signaling) were diluted 1/2,000. Bands were detected using enhanced chemiluminescence (ECL) (GE Healthcare, Little Chalfont, UK) according to manufacturer's instructions, and quantitation was performed digitally using the GE ImageQuant LAS4000 mini luminescent image analyser. Band signals were normalized relative to digitally quantified total protein using the Bio‐Rad stain‐free system according to manufacturer instructions. All results show the average ± SD (*n* = 3) of biological replicates generated from three independent cell cultures.

## 3 RESULTS

### 3.1 Experimental strategy

The effects of exposure to triclosan were studied using three human breast epithelial cell lines: the immortalized but nontransformed MCF‐10F cells (Soule et al., 1990), the MCF‐7 cancer cells, which are oestrogen responsive (Darbre & Daly, 1989; Shaw et al., 2006) but have low motility (Alamer & Darbre, 2018), and the MDA‐MB‐231 cancer cells, which are oestrogen unresponsive (Garcia et al*.,* 1992) but have higher motility (Alamer & Darbre, 2018). Effects were studied over a long‐term period of weeks of exposure on the basis that effects on migration and invasion were only noted previously in MCF‐7 cells after long‐term exposure to parabens (20 ± 2 weeks) (Khanna et al., 2014) or to aluminium (32–37 weeks) (Darbre et al., 2013) or to chemical UV filters (>20 weeks) (Alamer & Darbre, 2018) and in MDA‐MB‐231 cells to aluminium (20–25 weeks) (Bakir & Darbre, 2015) or to chemical UV filters (15 weeks) (Alamer & Darbre, 2018). For the long‐term exposures, each cell line was maintained under the same stock cell culture conditions in parallel, with the only difference being of addition or not of triclosan. In this way, the treated and control cultures were assayed at the same passage number and with no difference in culture medium except for the presence of triclosan. Triclosan was added at 10^−7^ M, on the basis of this having been the predetermined maximal concentration without adverse effects on the proliferation rate of any of the cell lines used here, and on the basis of being environmentally relevant in relation to measurements in human tissues (see Section 1).

### 3.2 Effect of triclosan on migration and invasion of MCF‐10F cells

**FIGURE 2:**
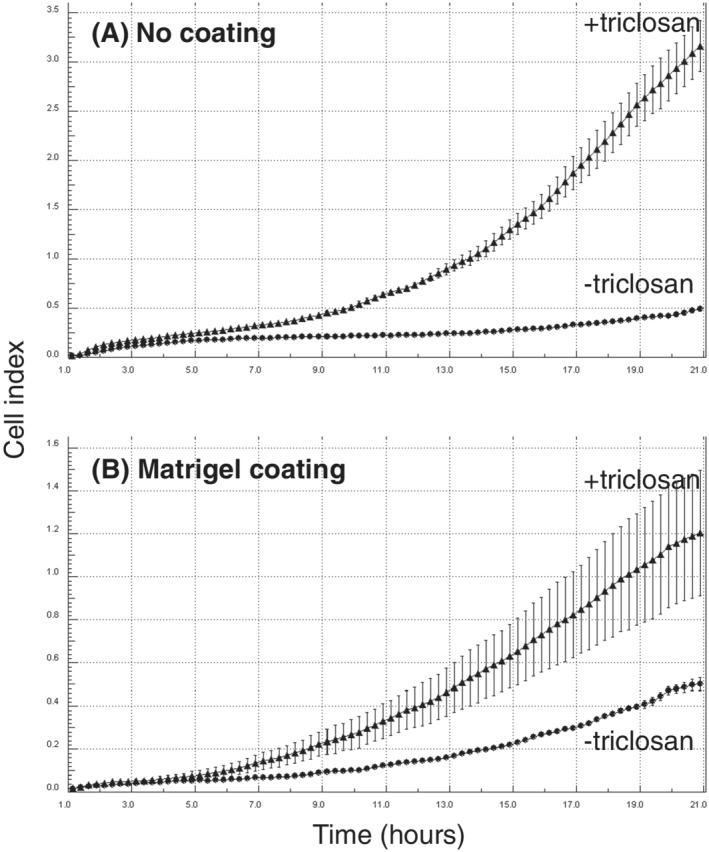
Effect of triclosan on migratory (A) and invasive (B) properties of immortalized, nontransformed MCF‐10F human breast epithelial cells as determined using xCELLigence technology with CIM‐16 plates uncoated (A) or coated with matrigel (B). Cell behaviour shown after prior cell culture for 28 weeks with (+triclosan) or without (−triclosan) 10^−7^ M of triclosan. Results are presented as the trace over time (1–21 h) for the average impedance values (cell index) ± SD of quadruplicate wells

Effects of exposure to 10^−7^ M triclosan on the migratory and invasive properties of MCF‐10F cells were investigated using xCELLigence technology (Figure 2). Migration of MCF‐10F cells is shown in Figure 2A after a prior 28 weeks of cell culture with or without 10^−7^ M triclosan. Time courses over 21 h showed that the rate of cell migration through uncoated membranes was increased in cells that had been exposed for a prior 28 weeks to triclosan as compared with control cells maintained for the same length of time without triclosan. Effects on the invasive properties of MCF‐10F cells were studied in parallel using matrigel‐coated membranes, and the rate of cell invasion was observed to increase in cells that had been exposed to the triclosan for 28 weeks as compared with those that had been exposed only as an ethanol control for the same length of time (Figure 2B).

### 3.3 Effect of triclosan on expression of E‐cadherin and β‐catenin in MCF‐10F cells

**FIGURE 3:**
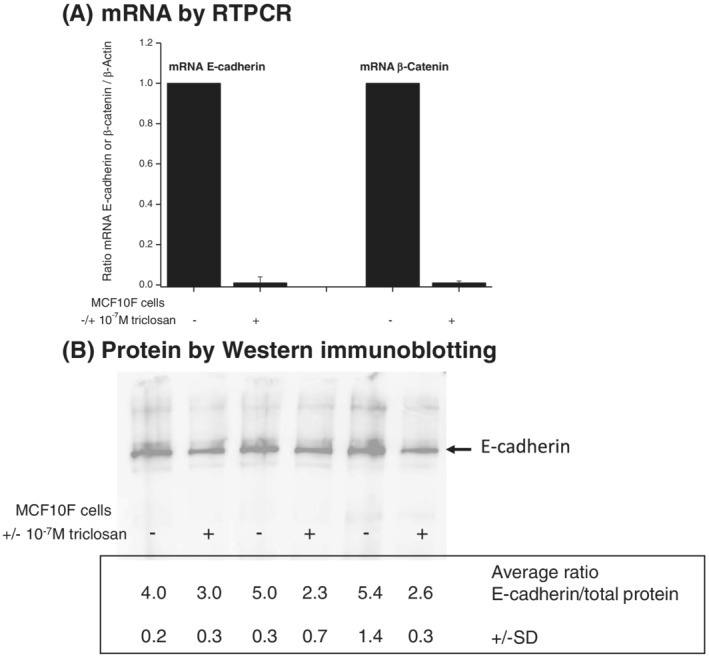
Levels of E‐cadherin and β‐catenin mRNA (A) as determined by RT‐PCR analysis and of E‐cadherin protein (B) as determined by western immunoblotting in immortalized, nontransformed MCF‐10F human breast epithelial cells following prior culture of cells with or without 10^−7^ M of triclosan for 30 weeks. RT‐PCR analyses (A) were normalized to β‐actin and controls without triclosan as described in Section 2: each analysis was carried out in triplicate on each of three independent biological replicates, and the values shown are the average ± SD for the three biological replicates. Immunoblots (B) show one representative gel of three biological replicates: values given are calculated average ratios of band intensity normalized to total protein ± SD for three replicate gels

One of the molecular mechanisms associated with increased cell motility involves downregulation of the transmembrane glycoprotein E‐cadherin followed by alterations to catenin signalling (Scheel & Weinberg, 2012). Reverse transcription–polymerase chain reaction (RT‐PCR) was used to investigate effects of long‐term (30 weeks) exposure to 10^−7^ M triclosan on levels of E‐cadherin mRNA and β‐catenin mRNA in the MCF‐10F cells (Figure 3A). Levels of both these mRNAs were dramatically reduced by the exposure to triclosan down to levels that were 1% of the levels in the control unexposed cells (Figure 3A).

Western immunoblotting was used to determine the effect of long‐term (30 weeks) triclosan exposure on levels of E‐cadherin protein. E‐cadherin was identified as a band of 135 kDa on the polyacrylamide gel against molecular weight markers. Figure 3B shows three biological replicates in which levels of E‐cadherin were reduced in cells following 30 weeks of exposure to 10^−7^ M concentrations of triclosan. The calculated ratios given underneath were of the scanned ECL values for E‐cadherin normalized to scanned values of total protein according to Bio‐Rad protocols, and the values and SDs shown were calculated from three technical replicates of running the same samples on three separate gels.

### 3.4 Effect of triclosan on migration and invasion of MCF‐7 cells

Effects of exposure to 10^−7^ M of triclosan were then investigated on the migratory and invasive properties of a second cell line, the MCF‐7 cells that are transformed cells with a relatively low motility (Alamer & Darbre, 2018). These cells are oestrogen responsive (Darbre & Daly, 1989; Shaw et al., 2006), but culture conditions of oestrogen deprivation were not used here. The MCF‐7 cells were grown long term as stock cultures in the presence of both insulin and 17β‐oestradiol in order to determine effects of triclosan without any other manipulations to the culture medium.

**FIGURE 4:**
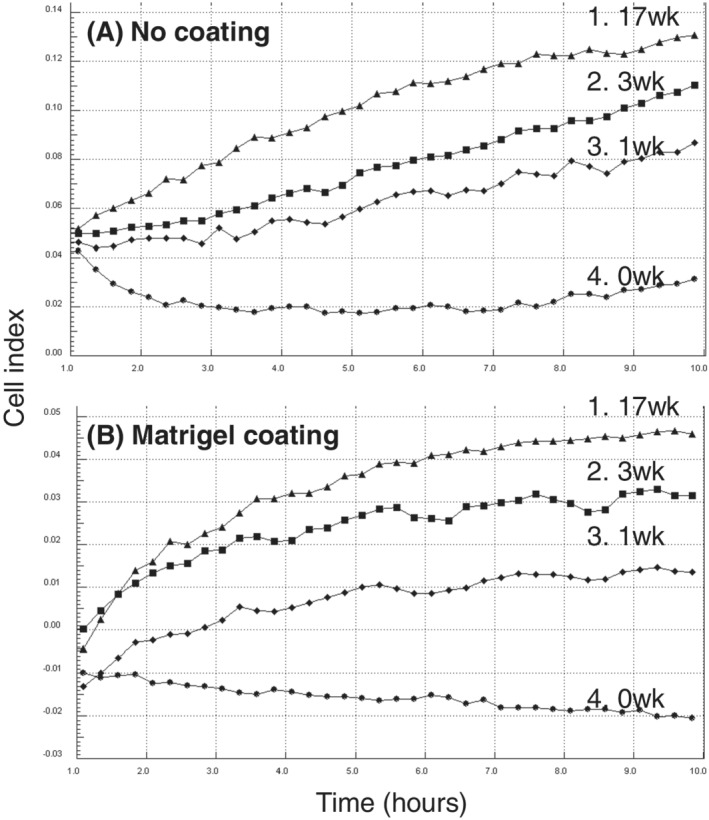
Effect of triclosan on migratory (A) and invasive (B) properties of MCF‐7 human breast cancer cells as determined using xCELLigence technology with CIM‐16 plates uncoated (A) or coated with matrigel (B). Cells were set up such that all these analyses were carried out on the same day with cells of the same passage number but after varying prior weeks (0–17) of cell culture with 10^−7^ M of triclosan as follows: 17 weeks with triclosan (1; 17wk), 14 weeks without triclosan followed by 3 weeks with triclosan (2; 3wk), 16 weeks without triclosan followed by 1 week with triclosan (3; 1wk), and 17 weeks without triclosan (4; 0wk). Results are presented as the trace over time (1–10 h) for the average impedance values (cell index) of quadruplicate wells

Traces of migration of MCF‐7 cells, as measured using xCELLigence technology, are shown in Figure 4A after varying weeks of cell maintenance with or without 10^−7^ M triclosan. For this experiment, MCF‐7 cells were established as four independent cell cultures grown to the same ultimate passage number for assay, as follows: (1) 17 weeks with triclosan, (2) 14 weeks without triclosan followed by 3 weeks with triclosan, (3) 16 weeks without triclosan followed by 1 week with triclosan, and (4) 17 weeks without triclosan. Time courses up to 10 h showed the rate of cell migration through uncoated membranes increased with length of prior exposure to triclosan for cells of the same passage number. Effects on the invasive properties of MCF‐7 cells were studied in parallel using matrigel‐coated membranes, and the rate of cell invasion was similarly increased with increasing length of prior exposure to triclosan (Figure 4B).

### 3.5 Effect of triclosan on expression of E‐cadherin and β‐catenin in MCF‐7 cells

**FIGURE 5:**
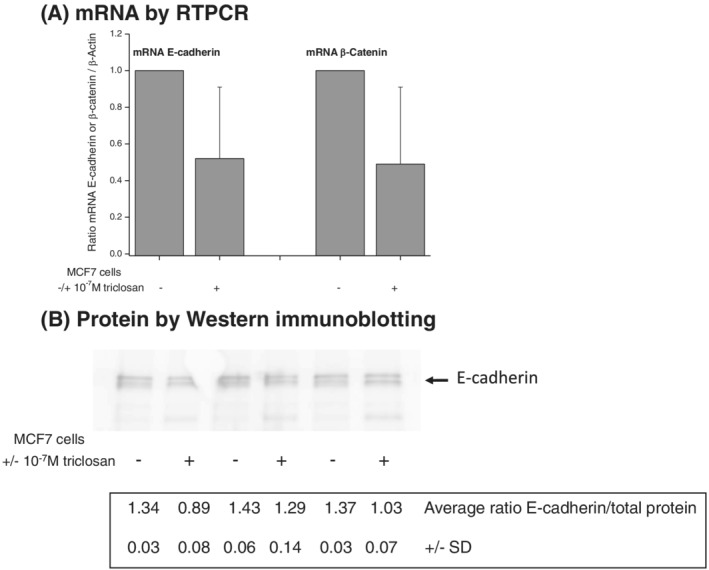
Levels of E‐cadherin and β‐catenin mRNA (A) as determined by RT‐PCR analysis and of E‐cadherin protein (B) as determined by western immunoblotting in MCF‐7 human breast cancer cells following prior culture of cells with or without 10^−7^ M of triclosan for 20 weeks. RT‐PCR analyses (A) were normalized to β‐actin and controls without triclosan as described in Section 2: each analysis was carried out in triplicate on each of three independent biological replicates, and the values shown are the average ± SD for the three biological replicates. Immunoblot (B) shows three biological replicates: values given are calculated average ratios of band intensity normalized to total protein ± SD

RT‐PCR was then used to investigate effects of long‐term (20 weeks) exposure to 10^−7^ M triclosan on levels of E‐cadherin mRNA and β‐catenin mRNA in the MCF‐7 cells (Figure 5A). Levels of both these mRNAs were reduced by exposure to triclosan (Figure 5A), although to a lesser extent than observed in the MCF‐10F cells (Figure 3A). Relative to control unexposed cells, cells treated with triclosan fell to an average level ± SD of 0.52 ± 0.39 for E‐cadherin mRNA and to 0.49 ± 0.42 for β‐catenin mRNA.

Western immunoblotting was used to determine the effect of long‐term (20 weeks) triclosan exposure on levels of E‐cadherin protein. Using the Cell Signaling antibody against E‐cadherin (3195; 24E10), we identified E‐cadherin in MCF‐7 cells as running as a double band at around 135 kDa on the polyacrylamide gel against molecular weight markers. The reason for a double band was not determined but was presumed to relate to breakdown forms of E‐cadherin previously visualized in ovarian cancer cells using this same antibody (Trillsch et al., 2016), and quantitation was carried out as a summation of the two bands. Figure 5B shows three biological replicates in which levels of E‐cadherin were reduced in cells following 20 weeks of exposure to 10^−7^ M concentrations of triclosan. The calculated ratios given below were of the scanned ECL values for E‐cadherin normalized to scanned values of total protein according to Bio‐Rad protocols, and the values and SDs shown were calculated from three technical replicates.

### 3.6 Effect of triclosan on migration and invasion of MDA‐MB‐231 cells

**FIGURE 6:**
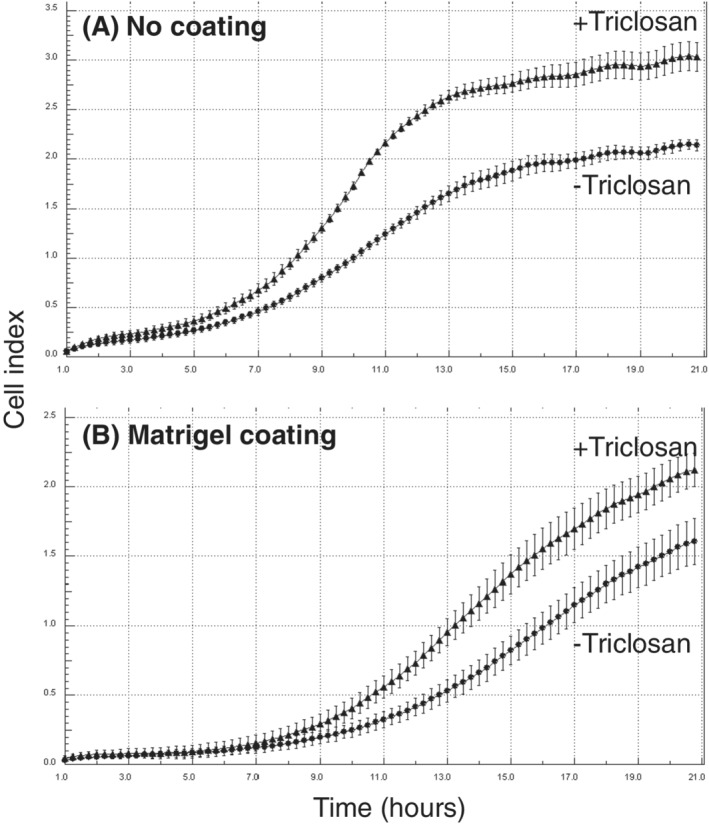
Effect of triclosan on migratory (A) and invasive (B) properties of MDA‐MB‐231 human breast cancer cells as determined using xCELLigence technology with CIM‐16 plates uncoated (A) or coated with matrigel (B). Cell behaviour shown after prior cell culture for 20 weeks with (+triclosan) or without (−triclosan) 10^−7^ M of triclosan. Results are presented as the trace over time (1–21 h) for the average impedance values (cell index) ± SD of quadruplicate wells

In order to determine whether triclosan could influence migratory and invasive properties of oestrogen‐unresponsive human breast cancer cells, the MDA‐MB‐231 cell line was used. This cell line has been reported as unresponsive to oestrogen for proliferation (Garcia et al., 1992), and we have never observed any increase in proliferation in response to 17β‐oestradiol in these cells in our laboratory. The MDA‐MB‐231 cells are generally more motile than the MCF‐7 cells (Alamer & Darbre, 2018). With the use of xCELLigence technology, migration of MDA‐MB‐231 cells is shown in Figure 6A after a prior 20 weeks of exposure with or without 10^−7^ M of triclosan. Time courses over 20 h showed the rate of cell migration through uncoated membranes was increased in cells that had been exposed for a prior 20 weeks to triclosan as compared with control cells maintained for the same length of time without triclosan. Effects on the invasive properties of MDA‐MB‐231 cells were studied in parallel using matrigel‐coated membranes, and the rate of cell invasion was also observed to increase in cells that had been exposed to the triclosan for 20 weeks as compared with those that had been exposed only as an ethanol control for the same length of time (Figure 6B).

MDA‐MB‐231 cells do not express E‐cadherin (Alamer & Darbre, 2018), and neither RT‐PCR nor western immunoblotting showed any measurable E‐cadherin in these cells.

## 4 DISCUSSION

These results have shown that long‐term exposures of three human breast epithelial cell lines to 10^−7^ M concentrations of triclosan result in increased migratory and invasive activity of the cells. These increases were observed after triclosan exposure in the untransformed breast epithelial cells (MCF‐10F) as well as in the breast cancer cells (MCF‐7, MDA‐MB‐231). These effects were observed in the oestrogen‐unresponsive (MDA‐MB‐231) as well as the oestrogen‐responsive (MCF‐7) human breast cancer cells. They were observed in the human breast epithelial cells irrespective of whether the cells possessed lower (MCF‐7 cells) or higher (MDA‐MB‐231 cells) intrinsic motility (Alamer & Darbre, 2018). In addition, the results revealed that triclosan exposure can result in increased penetration of the cells through more than one matrix, in that previous studies demonstrated an increase in migration through fibronectin (Lee et al., 2017), but these studies demonstrate increased invasion through matrigel. Finally, the results demonstrate that the increases in migration and invasion persist into the long term. A previous work has reported short‐term effects up to 72 h on migration and invasion of oestrogen‐responsive MCF‐7 human breast cancer cells after exposure to triclosan (Lee, Choi, & Hwang, 2017), and this study demonstrates that longer term exposure of many weeks continue to cause increased cell migration and invasion, reflecting the environmental reality that breast cells are exposed long term, not just for a few hours, to this widely distributed environmental chemical.

Although similar phenotypic changes were observed to migratory and invasive properties in all three of the human breast epithelial cell lines, the underlying molecular mechanisms appear rather to differ. In the cells that expressed E‐cadherin (MCF‐10F and MCF‐7), a reduction in expression of E‐cadherin was observed at both m‐RNA and protein levels, which would be consistent with a mechanism involving epithelial‐to‐mesenchymal transition (EMT) (Scheel & Weinberg, 2012). However, the MDA‐MB‐231 cells do not express E‐cadherin and so such a mechanism would not explain the observed increases in migration and invasion in these cells. Triclosan has been reported to possess oestrogenic activity (Ahn et al., 2008; Gee et al., 2008; Henry & Fair, 2013; Huang et al., 2014; Stoker et al., 2010), to bind to oestrogen receptors (Gee et al., 2008) and to enable increased migratory and invasive properties of the oestrogen‐responsive MCF‐7 human breast cancer cell line through an ER‐mediated mechanism (Lee, Choi, & Hwang, 2017). However, the results here showed that triclosan exposure also caused increased cell migration and invasion in oestrogen‐unresponsive (MDA‐MB‐231) human breast cancer cells as well as the oestrogen‐responsive (MCF‐7) cells. Furthermore, the effects of triclosan on migratory and invasive properties of MCF‐7 cells were studied here in the presence of already high concentrations (10^−8^ M) of 17β‐oestradiol. The study was performed this way so as not to alter any stock culture conditions of the cell line other than addition of triclosan. In order to study oestrogen action, it is necessary to grow the MCF‐7 cells under oestrogen‐deprived conditions, which involve removal of not only oestradiol but also other supporting growth factors including insulin. Previous short‐term studies (Lee, Choi, & Hwang, 2017) reported the increased motility of MCF‐7 cells under oestrogen‐deprived conditions, which was reversible with antioestrogen, demonstrating an oestrogen receptor‐mediated mechanism, but these studies demonstrate that triclosan can also influence cell motility in the presence of oestradiol, as would be the prevailing conditions in vivo in the human breast. With high levels of oestradiol already in the culture medium, the mechanism of altered MCF‐7 cell motility in these studies would be unlikely to result from a further oestrogenic stimulus. Therefore, although triclosan can increase cell motility through an oestrogen receptor‐mediated mechanism in oestrogen‐responsive cells such as MCF‐7 (Lee, Choi, & Hwang, 2017), its effects on breast cell motility are not limited to this mode of action even in these cells. Previously published studies have described the ability of triclosan to increase motility of H460 lung cancer cells through not only EMT but also a FAK/Akt and Rac‐1‐dependent mechanism (Winitthana, Lawanprasert, & Chanvorachote, 2014) and to increase motility of LNCaP prostate cancer cells by an androgen receptor‐mediated mechanism (Kim, Hwang, Shim, & Choi, 2015). Other endocrine‐disrupting chemicals, including bisphenol A, phthalate esters and 2,3,7,8‐tetrachlorodibenzo‐*p*‐dioxin (TCDD), have also been reported to show diverse ways of increasing motility of different cell types through EMT involving not only oestrogen receptor‐mediated mechanisms but other molecular pathways too (Lee, Hwang, & Choi, 2017). Studies with other ingredients of personal care products such as aluminium (Bakir & Darbre, 2015) and chemical UV filters (Alamer & Darbre, 2018) have shown an association between increased migratory and invasive properties of the MDA‐MB‐231 cells and increased secretion of some matrix metalloproteinases, which are extracellular proteases that aid in migration and invasion of cells. The extent to which metabolism of triclosan through phase 1 hydroxylation and/or phase II sulfation/glucuronidation reactions (Dann & Hontela, 2011; Weatherly & Gosse, 2017) might influence cellular effects remains unknown for these cell lines, but in view of the role for example of hydroxylation in oestrogenic action of polychlorinated organic compounds such as the PCBs (Ruiz, Ingale, Wheeler, & Mumtaz, 2016), this remains a relevant question in need of consideration. Finally, it also remains unknown as to whether mechanisms in longer term effects may differ from short‐term effects. Triclosan is known to cause many alterations to gene expression on microarray analysis (Li et al., 2020), and accumulation of these changes together with knock‐on consequences over the weeks may contribute to the final outcome.

Although triclosan levels have not been reported in human breast tissue per se, levels have been measured in human urine, human milk and human adipose tissue, which together suggest that the concentrations of triclosan used in these experiments at 10^−7^ M are environmentally relevant for the human breast. In a study of 2,517 human urine samples from the general U.S. population, triclosan was measured in 75% of the samples with a range of 2.4 μg/L (0.83 × 10^−8^ M) to 3,790 μg/L (1.3 × 10^−5^ M), a geometric mean of 13.0 μg/L (0.45 × 10^−7^ M) and a 95th percentile of 459 μg/L (1.6 × 10^−6^ M) (Calafat et al., 2008). With the use of human breast milk (*n* = 34), triclosan was measured in one third of samples in the United States with a range of 0–567 μg/L (2.0 × 10^−6^ M) and a median value of 18.4 μg/L (0.64 × 10^−7^ M) (Hines et al., 2015). Because breast is a fatty tissue, levels in adipose tissue are also relevant, and a study of human adipose tissues (*n* = 20) revealed triclosan concentrations with a geometric mean of 7.21 ng/g wet weight (equivalent to 0.25 × 10^−7^ M) and a maximum of 23.2 ng/g (equivalent to 0.8 × 10^−7^ M) (Wang et al., 2015), which are close to the concentration of 10^−7^ M used in these in vitro experiments.

Increased migration and invasion of cells are an essential component of metastatic tumour spread (Hanahan & Weinberg, 2011), and these results show that these features can be increased in human breast epithelial cells following long‐term exposure to triclosan at concentrations measurable in the human urine, milk and adipose tissue. Previous reports have demonstrated similar changes after exposure of human breast cancer cells to other chemical components of personal care products at environmentally relevant concentrations. This includes five paraben esters (Khanna et al., 2014), aluminium chlorohydrate (Bakir & Darbre, 2015; Darbre et al., 2013) and six chemical UV filters (Alamer & Darbre, 2018). Because all these compounds have been measured as present in human breast tissue, it must be assumed that exposures of the human breast in vivo would be to multiple compounds rather than single compounds. Additive effects on proliferation of human breast cancer cells have already been demonstrated when mixing multiple cosmetic chemicals with oestrogenic activity (Charles & Darbre, 2013), but it remains to be investigated as to whether similar additive effects might be measurable on cell migration/invasion and whether mixing multiple compounds might similarly enable effects to be observed at lower concentrations than when assayed alone. Because breast cancer mortality results from tumour spread rather than just increase in size of the primary tumour, the ability of these components of personal care products to increase cell migration and invasion deserves further consideration even if underlying molecular mechanisms differ between cell lines and between chemicals. Furthermore, if components of personal care products can act by increasing cell migration and invasion, then reduction in exposure could offer a strategy for reducing or preventing breast cancer spread.

## CONFLICT OF INTEREST

The authors have no competing financial interests.

## Data Availability

The data that support the findings of this study are available from the corresponding author upon reasonable request.
